# The Management of COVID-19-Related Coagulopathy: A Focus on the Challenges of Metabolic and Vascular Diseases

**DOI:** 10.3390/ijms241612782

**Published:** 2023-08-14

**Authors:** Mónika Szilveszter, Sándor Pál, Zsuzsánna Simon-Szabó, Orsolya-Zsuzsa Akácsos-Szász, Mihály Moldován, Barbara Réger, Lóránd Dénes, Zsuzsanna Faust, Mariana Cornelia Tilinca, Enikő Nemes-Nagy

**Affiliations:** 1 Clinic of Plastic Surgery, Mureș County Emergency Hospital, 540136 Târgu-Mureș, Romania; monikaszilveszter@yahoo.com; 2 Department of Transfusion Medicine, Medical School, University of Pécs, 7624 Pécs, Hungary; faust.zsuzsanna@pte.hu; 3 Department of Pathophysiology, Faculty of Medicine, George Emil Palade University of Medicine, Pharmacy, Science, and Technology of Târgu-Mureș, 540142 Târgu-Mureș, Romania; 4 Doctoral School, Faculty of Medicine, George Emil Palade University of Medicine, Pharmacy, Science, and Technology of Târgu-Mureș, 540142 Târgu-Mureș, Romania; szaszorsolya@yahoo.com; 5 Klinik für Suchttherapie, ZtP Winnenden-Haus der Gesundheit, 73525 Schwäbisch Gümund, Germany; m.moldovan@zpf-winnenden.de; 6 Department of Laboratory Medicine, Medical School, University of Pécs, 7624 Pécs, Hungary; reger.barbara@pte.hu; 7 Department of Anatomy and Embryology, Faculty of Medicine, George Emil Palade University of Medicine, Pharmacy, Science, and Technology of Târgu-Mureș, 540142 Târgu-Mureș, Romania; lorand.denes@umfst.ro; 8 Department of Internal Medicine I, Faculty of Medicine in English, George Emil Palade University of Medicine, Pharmacy, Science, and Technology of Târgu-Mureș, 540142 Târgu-Mureș, Romania; mariana.tilinca@umfst.ro; 9 Department of Chemistry and Medical Biochemistry, Faculty of Medicine in English, George Emil Palade University of Medicine, Pharmacy, Science, and Technology of Târgu-Mureș, 540142 Târgu-Mureș, Romania; eniko.nemes-nagy@umfst.ro

**Keywords:** COVID-19, viscoelastometry, hypercoagulable state, thrombosis, coagulopathy, venous thromboembolism, obesity, diabetes mellitus, cardiovascular diseases, stroke

## Abstract

The course of COVID-19 is highly dependent on the associated cardiometabolic comorbidities of the patient, which worsen the prognosis of coronavirus infection, mainly due to systemic inflammation, endothelium dysfunction, and thrombosis. A search on the recent medical literature was performed in five languages, using the PubMed, Embase, Cochrane, and Google Scholar databases, for the review of data regarding the management of patients with a high risk for severe COVID-19, focusing on the associated coagulopathy. Special features of COVID-19 management are presented, based on the underlying conditions (obesity, diabetes mellitus, and cardiovascular diseases), emphasizing the necessity of a modern, holistic approach to thromboembolic states. The latest findings regarding the most efficient therapeutic approaches are included in the article, offering guidance for medical professionals in severe, complicated cases of SARS-CoV-2 infection. We can conclude that severe COVID-19 is closely related to vascular inflammation and intense cytokine release leading to hemostasis disorders. Overweight, hyperglycemia, cardiovascular diseases, and old age are important risk factors for severe outcomes of coronavirus infection, involving a hypercoagulable state. Early diagnosis and proper therapy in complicated SARS-CoV-2-infected cases could reduce mortality and the need for intensive care during hospitalization in patients with cardiometabolic comorbidities.

## 1. Introduction

COVID-19 is an acute respiratory infection caused by the severe acute respiratory syndrome virus SARS-CoV-2. Risk factors, such as age, male gender, associated chronic diseases, and/or altered metabolic states determine the severity of this condition, leading to a significant burden for intensive care units (ICUs) [1,2,3,4].

The continuously rising prevalence of type 2 diabetes mellitus (T2DM) worldwide, as well as obesity, further increases the burden on ICUs, due to the greater risk of these patient groups developing severe and critical COVID-19 symptoms. The clinical course of COVID-19 is aggravated even more by the presence of multiple comorbidities. Additionally, a higher prevalence of T2DM and vasculopathies has been reported in COVID-19 patients [5,6].

SARS-CoV-2 infects the host by binding to the angiotensin-converting enzyme 2 receptor (ACE2-R) expressed on different cell membranes. Some studies suggest that overexpressed ACE2-R in diabetic patients increases the risk of infection with the virus [7,8,9].

In chronic disease, constant and prolonged inflammation is present, with slightly increased synthesis of several cytokines, some of which have a predictive role in the development of type 2 diabetes (interleukins IL-1β, IL-6) [10,11]. Chronic low-grade inflammation also contributes significantly to the pathogenesis of atherosclerosis, due to the effects of tumor necrosis factor-alpha (TNF-α) and interleukin-10 (IL-10) [12].

Typically, COVID-19 causes a procoagulant state, through endothelial dysfunction and the malfunction of leukocytes and platelets, systemic inflammation, and the activation of several inflammatory pathways. This pattern has been termed COVID-19-associated coagulopathy (CAC), and can result in deep vein thrombosis (DVT), pulmonary embolism (PE) and stroke, further increasing the risk of mortality. SARS-CoV-2-infected patients with preexisting comorbidities are at risk of even higher mortality rates [13,14].

Hemostasis is the balanced interplay of the prothrombotic and antithrombotic processes. Conventional plasma-based hemostasis assays are mostly used for the assessment of the bleeding and coagulation risk in a surgical, traumatized, or intensive care patient. These tests carry limitations, as well, due to their ability to assess isolated diseases related to clotting factors, or the effect of an anticoagulant therapy.

Viscoelastic hemostatic tests were introduced in the 1950s, and were primarily used intraoperatively, as point-of-care methods. The utility of viscoelastic tests is to provide information about the global activity of hemostasis, including platelet aggregation effectiveness, thrombin generation, clot formation, and clot lysis. The duration of the measurements is 60 min, although, in most cases, valuable information is obtained after the first 20 min of the test, providing the possibility of early, individualized therapy for the patient [15,16]. According to Tyler et al., point-of-care viscoelastometric tests should be included for all patients requiring a massive transfusion, whether surgical or non-surgical, and whether trauma-related or not. In the USA, viscoelastometric test training is recommended for general surgery residents, and a further expansion of this method is expected in the near future [17]. Significant differences in the levels of inflammatory biomarkers and metabolic parameters in moderate or severe COVID-19 patients (with diabetes/obesity) have been described: in contrast to infected subjects without these comorbidities, the diabetic/obese patients with severe COVID-19 presented a significantly increased leukocyte count, erythrocyte sedimentation rate (ESR), C-reactive protein (CRP), D-dimers, and serum glucose concentration [18]. A comprehensive study performed in Romania revealed that elevated inflammatory markers were independent predictors of poor outcomes for all SARS-CoV-2-infected patients. In the same study, the authors observed that patients with associated cardiac and renal diseases, peripheral arteriopathy, obesity, dyslipidemia, malignancies, and tobacco use were predisposed to a higher mortality [19].

In a pilot study, eosinopenia and lymphopenia were associated with a severe outcome in COVID-19 [20].

The unfortunate coexistence of a proinflammatory and prothrombotic status in SARS-CoV-2 infection in some patients is considered a key mechanism in the progression of severe and critical cases of COVID-19, with the severity further increasing in patients with an existing proinflammatory and prothrombotic status due to recorded comorbidities. The recommended therapies for the different patient groups, based on their risk of developing serious COVID-19 conditions, aim to reduce inflammatory processes and viremia, and mitigate the thrombosis risk.

The authors aimed to synthesize the population-specific impact of comorbidities, such as diabetes, obesity, and cardiovascular disease, on COVID-19 outcomes, and to highlight the difficulties in diagnosis, and the challenges in the management of COVID-19 patients with comorbidities. Many observational studies and research reports were published during the pandemic, mostly in English, but presumably also in other languages, so the search criteria included not only papers published in English, but also in Spanish, German, Hungarian, and Romanian.

## 2. Materials and Methods

A literature search was conducted between 10 December 2022 and 10 June 2023, using PubMed, Embase, Cochrane Library, and Google Scholar, restricting the results to open-access articles written in English, Hungarian, Romanian, German, or Spanish, and published between 1 January 2020 and 1 June 2023. A combination of the following keywords and phrases was used in the five different languages: “COVID-19 coagulopathy therapy”, “COVID-19 thrombosis therapy”, “COVID-19 thrombosis treatment”, “cytokines”, “obesity”, “type 2 diabetes”, “anticoagulation”, “hypercoagulable state”, “SARS-CoV-2 induced hypercoagulability”, “critically ill COVID-19 viscoelastometry”, “critically ill COVID-19 thromboelastometry”, “viscoelastic tests and COVID-19”, and “vasculopathy and COVID-19”.

After performing the search, the authors screened the articles based on the title and abstract, and a further selection was also performed after the full-text assessment of the remaining articles. The authors also selected secondary sources cited by recent systematic reviews or meta-analyses, including the most relevant research articles.

## 3. Proinflammatory and Procoagulant Effects of COVID-19

During SARS-CoV-2 infection, inflammation plays a significant role in the development of the disease. Studies have demonstrated the release of numerous inflammatory cytokines and chemokines in COVID-19. The innate immune system is instrumental in the immune response against pathogens, and while the production of proinflammatory cytokines is crucial, excessive activation of these cytokines can lead to widespread damage in certain cases of COVID-19. Several cytokines, including IL-2, IL-6, TNF-α, IFN-γ, MIP (macrophage inflammatory protein), and MCP-1 (monocyte chemoattractant protein-1), are prominently present in severely ill patients. Angiotensin II (AngII) triggers the activation of nuclear factor kappa-B (NF-κB), resulting in hyperinflammation, primarily through the increased synthesis of IL-6 and IL-1β, leading to an enhanced transcription of proinflammatory cytokines. These interleukins exhibit elevated levels in severe cases of COVID-19 [21].

IL-6, a proinflammatory cytokine, not only stimulates the release of other cytokines, but also activates immune cells, playing an important role in the systemic inflammation which is common in severe COVID-19 cases [22]. IL-6 and IL-1α play a major role in connecting the inflammatory reaction and the blood-clotting system. During inflammation, macrophages release tissue factor in response to IL-6 [23]. The overexpression of IL-6 and its receptor in COVID-19 leads to the hyperactivation of endothelial cells. This hyperactivation will release a substantial amount of tissue factor, contributing to infection-induced coagulopathy, which is involved in the mechanism of thrombocytopenia, while the cytokine storm induces thrombocytosis. IL-6 is also involved in the production of certain coagulation factors, such as fibrinogen and factor VIII. Furthermore, IL-6 acts on the endothelium to enhance the synthesis of vascular endothelial growth factor (VEGF), leading to vascular hyperpermeability and hypotension [24]. Other cytokines, including TNF-α, IFN-γ, and IL-1β, have also been involved in the intense cytokine release described in COVID-19 patients, contributing to the proinflammatory state and hypercoagulability. These cytokines are present on different cell types, such as activated platelets, monocytes, and endothelial cells during the proinflammatory phase. IL-1α not only activates the cascade of inflammation in thrombo-inflammatory pathologies, but also plays a key role in thrombogenesis, by recruiting granulocytes, prolonging the clot-lysis time, and increasing the platelet activity [25]. Conversely, together with TNF-α, IL-1 is the most important mediator of the suppression of the endogenous coagulation cascade [23].

## 4. Low-Grade Chronic Inflammation and Its Consequences in COVID-19 Patients with Comorbidities

The precise mechanisms and interactions between cytokines in individuals with diabetes mellitus, other chronic comorbidities, and vascular and metabolic diseases who are infected with SARS-CoV-2 are currently the subject of active research.

Adipose cells release cytokines, and prolonged high blood sugar levels have an immunomodulatory effect, contributing to the maintenance of chronic low-grade inflammation. Obese people are more prone to develop insulin resistance and type 2 diabetes, and insulin resistance is also commonly seen in diabetic patients. Type 2 diabetes mellitus leads to complications affecting both the microvascular and macrovascular systems, particularly impacting endothelial cells [26,27]. Endothelial dysfunction, in turn, induces a prothrombotic state often accompanied by chronic inflammatory processes. Obesity and cardiovascular comorbidities in patients with COVID-19 significantly increase the risk of severe and/or critical symptoms, prolonged hospitalization, the need for intensive therapy, mechanical ventilation (both non-invasive and invasive), and higher mortality rates [28,29,30,31]. Additionally, the combination of these comorbidities further escalates the risk of morbidity and mortality. Endothelial dysfunction, when exacerbated by the cytokine storm triggered by SARS-CoV-2, emerges as the primary cause of death [32,33,34]. Viral toxicity also contributes to the severity of COVID-19. Particularly in vulnerable patients, virus-induced pathomechanisms, such as a prothrombotic state, cytokine storm, immune system dysregulation, and inflammation are underlying factors in the critical manifestation of COVID-19. These pathomechanisms are more frequently observed in obese and diabetic patients, and in those with multiple comorbidities. Deceased COVID-19 patients exhibited significantly higher levels of D-dimers and fibrin degradation products (FDPs) compared to the surviving COVID-19 group. In addition, disseminated intravascular coagulopathy (DIC) was found to be more common in the group of deceased COVID-19 patients compared to survivors [7,35].

In elderly obese patients with COVID-19, elevated C-reactive protein (CRP), ferritin, and interleukin 6 (IL-6) levels were strongly associated with critical disease. Type 2 diabetes mellitus was more prevalent in severe-COVID-19 patients compared to less-severe cases and the general population [36]. After infection with SARS-CoV-2, some non-diabetic subjects showed hyperglycemia and significantly higher interleukin-8 (IL-8) concentrations compared to the normoglycemic COVID-19 group [37]. COVID-19 patients with diabetes mellitus (DM) had significantly higher mortality rates compared to non-diabetic COVID-19 subjects, especially those with poorly controlled glucose levels, who were at the highest risk of complications [27,38].

## 5. Assessment of Hemostatic Activity in COVID-19

The hemostatic activity of COVID-19 patients is frequently hindered, and the pathomechanisms triggered by the SARS-CoV-2 infection result in a prothrombotic state [39]. Performing conventional or point-of-care hemostatic assays enables clinicians to assess and correct the underlying cause of the prothrombotic state. The activated partial thromboplastin time (APTT), prothrombin time (PT), thrombin time (TT), fibrinogen, and fibrinolysis parameters (such as D-dimers), were useful in COVID-19 patients for thrombosis risk assessment, and also for therapy.

Hemostatic abnormalities of COVID-19 patients include increased D-dimer levels and hyperfibrinogenemia [40]. Several scientific papers suggest that the severity of COVID-19 is associated with prolonged PT and TT, and a trend toward a shortened APTT [13,41]. Compared to the sepsis-related DIC, the D-dimer levels of COVID-19 patients are significantly higher. Furthermore, as fibrinogen is an acute-phase protein, excessive inflammation leads to markedly increased fibrinogen levels, as well. The D-dimer concentration is in a positive correlation with poorer outcomes of COVID-19 cases. In asymptomatic COVID-19 cases, high D-dimer and fibrinogen levels were associated with a high risk of hospitalization [42,43]. Further laboratory anomalies include thrombocytopenia [44] or thrombocytosis [45].

A higher FVIII and higher Von Willebrand factor (VWF) are also specific features of COVID-19-associated coagulopathy [46]. Their elevation in patients with ongoing inflammatory processes is expected, with both being acute-phase reactants. Elevated FV concentrations were reported in severe COVID-19 cases, and these were associated with a high risk of venous thromboembolic events [47].

Conventional hemostatic assays are important elements of coagulopathy diagnosis in COVID-19, based on which the clinicians decide the patient’s therapy. The measurement of procoagulant factors is often expanded with laboratory assays assessing thrombophilia, whether it is inherited or acquired. These assays most commonly include protein C (PC)-, protein S (PS)-, and antithrombin (AT)-deficiency testing [48]. Acquired thrombophilias are also often related to viral infections, and SARS-CoV-2 infection does not seem to be an exception. In severe COVID-19 cases, PS deficiency has been described in 20% of the subjects [49], while other studies reported near-threshold low levels of AT, PC, or PS [50].

Viscoelastic hemostatic tests are based on the distinctive property of blood, wherein its viscosity undergoes changes parallel to the formation of the blood clot during coagulation. Moreover, the resulting blood clot has elastic properties. The method, known as the viscoelastic test, is named after the combined assessment of two physical properties: the viscosity and elasticity. Viscoelastic tests are performed on whole-blood samples and, therefore, measure the hemostatic activity of the patient, including their cells, platelets, and plasma proteins, that can assess their hypocoagulation or hypercoagulation status.

In clinical practice, the viscoelastic method provides valuable data regarding the initiation of coagulation processes, blood clot development and firmness, clot lysis, and fibrinolysis effectiveness. The method includes several parameters, e.g., the clotting time (CT), clot formation time (CFT), maximum clot firmness (MCF), maximum lysis (ML), and lysis time (LT) [51]. The viscoelastic test is an ex vivo model of certain coagulation pathways, which analyze extrinsic (EX-test), or intrinsic (IN-test) pathways, or intrinsic and common pathways, and there are possibilities for the assessment of coagulation with the inhibition of certain elements (the FIB-test eliminates the effect of thrombocytes in the coagulation process). For more details, we refer you to Table 1.

In patients with COVID-19, the EX-test, IN-test, FIB-test, and TPA-test (EX-test with additional fibrinolysis initiation with the addition of tissue plasminogen activator—tPA) provide the most useful information on the coagulation status. In COVID-19, the EX-test and IN-test may assess the relative procoagulation (shortened CT) or hypercoagulation (increased MCF). An up-regulated platelet aggregation may also be diagnosed if, when comparing the FIB-test and EX-test, the MCF is more increased during the latter than in the case of the FIB-test. The TPA-test is useful in the assessment of antifibrinolytic dysfunction (fibrinolysis shutdown), with the test resulting in reduced ML or prolonged LT following tPA deficiency, plasminogen deficiency, or an increase in the plasminogen activator inhibitor-1 [52].

ROTEM is frequently used in polytrauma (patients with massive bleeding who require massive transfusions [53]), and can also be a valuable tool for the diagnosis of hemostatic disorders, and therapy-efficiency monitoring in antiplatelet or anticoagulant treatment. The SARS-CoV-2 pandemic further expanded the utility of the method, enabling a faster diagnosis of certain coagulation disorders in COVID-19 patients [54].

The fibrinolytic activity of a critically ill COVID-19 patient is impaired. Hypofibrinolysis occurs frequently in these cases, and it can be diagnosed using a visco-elastometric method, generally measuring a lysis time (LT) longer than 393 s. The hypofibrinolytic status of COVID-19 patients has also been named by several authors as ”fibrinolysis shut down” [55,56]. This mechanism could be treated via thrombolytic therapy, using recombinant tissue plasminogen activators.

## 6. Management of COVID-19 Patients

### 6.1. The Therapeutic Approach of COVID-19

**Figure 1:**
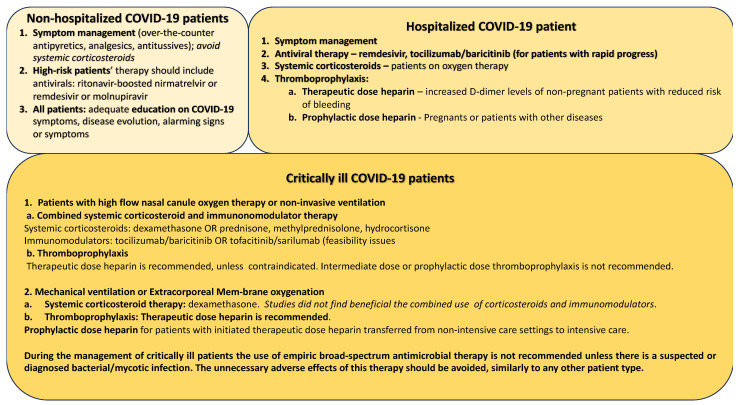
The management of COVID-19.

The special recommendations suggest a different therapeutic approach based on patient risk stratification, considering antiviral therapy, inhalation, or systemic corticosteroids, and thromboprophylaxis. Thus, the management of COVID-19 patients considers the individual characteristics of the cases, including the age, comorbidities, COVID-19 symptom severity, and risks for hospitalization, critical illness, and mechanical ventilation necessity. The therapeutic approach and drug recommendations are shown in Figure 1, based on the latest National Institutes of Health guidelines [57,58].

The management of outpatients infected with SARS-CoV-2 includes the revised therapy of their eventual comorbidities and chronic illnesses, with further recommendations and, in high-risk subpopulations, the available antiviral therapies should also be prescribed.

In several countries, ivermectin, an antiparasitic drug, has been prescribed for SARS-CoV-2-infected patients, based on the positive results of a research on the ability of ivermectin to inhibit the replication of SARS-CoV-2 in cell cultures [59]. However, pharmacokinetic and pharmacodynamic studies revealed that a 100-fold increase in the plasma concentration of ivermectin would be necessary to achieve this antiviral effect [60]. Furthermore, human clinical trials revealed that, compared to a placebo or standard care, ivermectin could not significantly benefit COVID-19 patients, and had minimal effect. Thus, after concluding these trials, the use of ivermectin for the therapy of COVID-19 is not recommended [61,62,63]. Outpatients with a high risk of developing severe COVID-19 symptoms should receive antiviral agents that include ritonavir-boosted nirmatrelvir or remdesivir [64,65]. In the case of hospitalized patients, intravenous antivirals would be appropriate (tocilizumab). These antiviral agents may be available in developed countries but, in underdeveloped areas, the patient’s access to these therapies may be limited or absent. Moreover, the expensive therapy in severe COVID-19 cases may also limit the possibility of acquiring the latest medications, even in a developed country.

The combined use of the available antiviral agents is not fully understood, and the available data on the combined use of antiviral therapies is limited [66].

Antiviral therapy further includes anti-SARS-CoV-2 monoclonal antibodies (mAbs). According to the latest clinical data, the efficacy of anti-SARS-CoV-2 mAbs depends on the subvariant of the SARS-CoV-2; moreover, Omicron and its sub variants show an increased resistance to this therapy type. Thus, the use of anti-SARS-CoV-2 mAbs is not recommended [67,68,69,70,71,72,73].

#### Multisystem Inflammatory Syndrome (MIS)

A special condition in COVID-19 occurs in patients with minimal respiratory symptoms and confirmed SARS-CoV-2 infection, in association with extreme systemic inflammation, markedly elevated C-reactive protein levels, ferritin, D-dimers, cardiac enzymes, creatinine, and liver enzymes, along with various other symptoms, including fever and shock. Furthermore, neurologic, cardiac, and gastrointestinal diseases are also frequently present in these cases. The above-mentioned signs and symptoms have been referred to as multisystem inflammatory syndrome in adults (MIS-A) [74,75,76].

According to the Centers for Disease Control and Prevention in the United States of America, the case definition of MIS-A is as follows: patients at least 21 years old, who were hospitalized for at least 24 h or whose illness concluded with fatality, and who met the clinical and laboratory criteria included below during the first 3 days of hospitalization. Alternative diagnoses for the condition should also be excluded.

The primary criteria for MIS-A include severe cardiac disease or rash, accompanied by nonpurulent conjunctivitis. The secondary criteria include new-onset neurologic disorders (encephalopathy, seizures, etc.), shock or hypotension not related to medical therapy, thrombocytopenia, abdominal pain, or vomiting or diarrhea. The laboratory criteria are based on the presence of SARS-CoV-2 infection concomitantly with extremely high levels of inflammatory biomarkers (at least two out of CRP, ferritin, erythrocyte sedimentation rate, procalcitonin, and IL-6) [77].

The management of MIS-A involves supportive care, immunosuppression, anticoagulant therapy, and inotropes. Biologic therapies targeting IL-6 and IL-1 have also been reported as being in use for cytokine storm, but the lack of evidence and randomized control trial data on their efficacy in the management of MIS-A means that the therapy is based on the expertise and protocols for Kawasaki disease (KD). A suggested approach could be the use of colchicine, an antiinflammatory drug, which has been reported to have clear cardiovascular benefits in pericarditis and myocardial infarction, and may hasten the resolution of cardiogenic shock in MIS-A. The long-term outcome of recovered MIS-A is uncertain because of the cardiovascular sequels [78].

The United States Centers for Disease Control and Prevention (CDC) and the World Health Organization (WHO) have defined multisystem inflammatory syndrome in children (MIS-C) as an acute condition, which includes what the Royal College of Pediatrics and Child Health has termed as pediatric multisystem inflammatory syndrome temporally associated with COVID-19 (PIMS-TS), and which has symptoms similar to those of Kawasaki disease (KD) and toxic shock syndrome (TSS) [77,79]. The systemic review conducted between December 2019 and July 2021 by M.O Santos at al. summarized a total of 98 articles from 18 countries, and concluded that a differential diagnosis between KD- or TSS-related MIS-C, and MIS-C in COVID-19 was a real challenge for physicians, due to the clinical resemblance; however, there were some particularities that were characteristic only of COVID-19-related MIS-C: the mean age of 9 years; severe abdominal pain due to ascites and mesenteric lymphadenitis, which needed advanced imaging and surgical investigations; and the presence of gastrointestinal symptoms, which occurred more often than in adults. The developed cardiac dysfunction progressed rapidly, and admission into the ICU and the management of hypotension and shock was necessary in some cases, while less-severe cases developed mild and transient coronary artery dilation [79]. There were several theories regarding the development of MIS-C related to COVID-19, and some findings suggest that the hyperinflammatory syndrome most likely occurs due to a postinfectious cytokine storm, rather than as a result of direct cell injury caused by intracellular SARS-CoV-2 replication. Riollano-Cruz et al. demonstrated that an elevated IL-6 level is present in MIS-C in comparison to COVID-19, and IL-1 elevation is absent in contrast to KD, where increased IL-1 levels play an important role in hyperinflammation [80]. According to the systemic review, the management of MIS-C related to COVID-19 involved the WHO protocols of KD and shock, and were represented by intravenous immunoglobulin, antiplatelet, or anticoagulant drugs, and the administration of steroids and biological immunomodulators. Inotropic agents, fluid resuscitation, and ventilatory support were provided when indicated (in a minority of cases) and, in severe cases, extracorporeal membrane oxygenation (ECMO) was used. If applicable, the initial broad-spectrum antibiotic administration was suspended after confirmation of MIS-C related to COVID-19 [79].

The mortality of MIS-A was 5–7% overall, threefold higher than MIS-C, and the predominant cause was reported to be refractory shock, myocarditis, vasculitis, and endothelitis [78].

### 6.2. The Therapeutic Approach to COVID-19 Coagulopathy

According to several studies, elevated D-dimer and fibrinogen levels are important predictors of mortality; thus, anticoagulation has been proposed in these cases. Heparin can be a good option for thromboprophylaxis, except in patients with antithrombin III (AT-III) deficiency, who use direct thrombin inhibitors, such as argatroban, which can be a better choice for systemic anticoagulation [33].

The measurement of serum albumin and AT-III activity is recommended in the algorithm of evaluating SARS-CoV-2-infected patients, and supplementation of these parameters at low levels has been shown to be beneficial. Albumin deficiency is involved in the development of edemas and circulatory failure (major mortality factors), while a low AT-III activity increases thromboembolic complications [81]. Deep vein thrombosis and pulmonary embolism are the most frequent thromboembolic conditions in COVID-19, with the affected patients showing a poor outcome and high mortality rate [19]. Medication of the most common cardiovascular disease, arterial hypertension, is an important concern during the pandemic [82].

The requirement of unusually large doses of heparin to achieve therapeutic values of APTT raises the suspicion of heparin resistance. Patients’ resistance to heparin therapy may occur in different circumstances: heparin pseudo-resistance, antithrombin III (ATIII) deficiency, low heparin concentration, and COVID-19-related heparin resistance.

Heparin pseudo-resistance is characterized by the temporary inability to detect the effect of heparin after the administration of the therapeutic dose. This occurs due to higher levels of FVIII and fibrinogen, which lead to a shortened APTT and, in certain inflammatory processes, this artificially low APTT value masks the effectiveness of the administered heparin. Thus, clinicians may misinterpret the results, believing that heparin therapy is ineffective, although it is exerting the desired effect [83].

Real heparin resistance is caused by a deficiency of ATIII. Heparin acts by binding to ATIII, and the lack of the latter leads to true heparin ineffectiveness. ATIII deficiency could occur due to consumption or lack of synthesis, and may be related to numerous pathological states, including liver diseases, acute thrombosis, and DIC.

Severe systemic inflammatory processes may hinder the binding of heparin to ATIII, causing a low heparin concentration. Acute-phase proteins and systemic inflammation enhance the synthesis of proteins that can bind to heparin, such as PF-4 (platelet factor 4).

The heparin resistance observed in COVID-19 patients is based on a combination of several factors. This phenomenon occurs due to increased levels of von-Willebrand factor and antiphospholipid antibodies, or based on the mechanism described in the case of pseudo-heparin resistance or low antithrombin III, or a combination of these mechanisms [84].

The management of heparin resistance consists of increased heparin dose administration, or use of alternative therapies, such as ATIII supplementation or the administration of direct thrombin inhibitors [85].

In the case of COVID-19 patients with elevated D-dimer levels, requiring conventional oxygen therapy without an increased bleeding risk, the administration of a therapeutic dose of heparin is recommended. Those patients, with different criteria, or pregnant, should receive a prophylactic heparin dose [86,87,88].

Furthermore, for patients starting therapeutic heparin prophylaxis in non-intensive care settings, who are then transferred to an intensive care unit, it is recommended to up-dose to therapeutic heparin if there is an indication for the administration of heparin therapy. Therapeutic or intermediate dose venous thromboembolism (VTE) prophylaxis for critical COVID-19 patients is not recommended, apart from being included in a clinical trial [89,90].

The benefits and drawbacks of routine screening for VTE in COVID-19 patients have not been established, so healthcare professionals should determine the frequency of such examinations. Nevertheless, COVID-19 patients experiencing a rapid deterioration in their condition, or developing cardiovascular or neurological complications should undergo routine VTE screening. In suspected cases of thromboembolism, therapeutic anticoagulation should be initiated, even without diagnostic imaging confirmation. Critically ill COVID-19 patients requiring life-support therapies, such as ECMO, NIV, and hemodialysis should be managed with anticoagulants, similar to other patient groups.

Several studies investigated the incidence of VTE in COVID-19 patients, and reported variable results. Notably, clinical data demonstrated that ultrasound screening has led to more frequent VTE diagnoses, compared to laboratory and clinical screening, with the results showing a four-fold difference in favor of ultrasound screening [91,92,93].

Compared to critically ill septic patients, the incidence of VTE in the COVID-19 patient group was similar; after thromboprophylaxis or antithrombotic therapy, the VTE incidence declined in all patient groups [94,95].

The therapeutic or prophylactic administration of oral anticoagulants is not recommended; instead, a prophylactic dose or therapeutic dose of heparin should be administered for VTE prophylaxis or the prevention of COVID-19 progression.

The interaction between antithrombotic agents and the newly introduced ritonavir-boosted nirmatrelvir, an antiviral combination that strongly affects the cytochrome P450 (CYP450) liver enzyme, can significantly alter the effectiveness of other co-administered drugs.

It is important to continue antithrombotic therapy for COVID-19 patients with preexisting comorbidities if it was already initiated prior to SARS-CoV-2 infection. However, in the case of a significant bleeding risk or of other hemostasis disorders, it is advisable to modify the therapy. Antiplatelet therapy prior to COVID-19 disease has been shown to reduce the overall mortality in hospitalized patient groups. However, the bias of these retrospective cohort studies cannot be fully removed from these studies [96,97,98,99].

Other clinical trials concluded that additional aspirin therapy in COVID-19-hospitalized patients did not affect mortality, and increased the bleeding risk of the patients [100]. Similar results were obtained in another trial regarding the additional use of purinergic P2Y12 receptor inhibitors [101]. According to the results of these studies, there is an inconsistency regarding the beneficial effects of these additional therapies; therefore, the initiation of antiplatelet therapies should not be used to treat patients with COVID-19 unless they are already receiving those drugs to treat pre-existing conditions.

### 6.3. Therapy of Comorbidities in COVID-19 Patients

COVID-19 has the potential to progress to severe conditions, such as hypoxemic respiratory failure, acute respiratory distress syndrome (ARDS), septic shock, cardiac dysfunction, thromboembolism, liver and/or kidney dysfunction, central nervous system disease, or the worsening of comorbidities. Furthermore, weeks or months after SARS-CoV-2 infection, adults may experience multisystem inflammatory syndrome, potentially resulting in critical illness (MIS-A or, in a child, MIS-C). Thus, the therapy for COVID-19 should also include the treatment of comorbidities. The general recommendations regarding the management of COVID-19 patients with comorbidities are shown in Table 2.

Diabetic patients frequently use oral antidiabetic therapy. Among these, metformin has shown potential, based on several in vitro studies, to be effective in the therapy of COVID-19, due to its antiviral, antithrombotic, and antiinflammatory activities [102,103,104,105].

Observational studies concluded that COVID-19 patients on metformin therapy were at a lower risk of progressing to severe and critical illness; therefore, these patients should continue their treatment [106,107,108].

In the case of metformin therapy, the side effects of this drug should also be considered (nausea, vomiting, diarrhea, headache), including the rarely occurring lactic acidosis, especially in high-risk patient groups (elderly, obese, metabolic diseases, cardiac dysfunction, hepatic or renal function impairment, excessive alcohol use) [109].

Additionally, vitamin C, vitamin D, and zinc and magnesium could also be part of the COVID-19 patients’ treatment, to improve the immune response in these subjects [110,111,112,113].

Currently, there is a lack of clinical data regarding the effectiveness or the utility of supplement administration. Notably, patients with comorbidities have reduced concentrations of several vitamins and trace elements; therefore, it sounds reasonable to supply COVID-19 patients with comorbidities with the vitamins and trace elements they lack [114]. Further data are needed to clarify whether the high-dose parenteral administration of certain vitamin supplements is recommended or not.

Community-based studies have revealed the efficacy of molnupiravir and paxlovid in the treatment of high-risk COVID-19-infected patients, including diabetic, obese, elderly subjects, and those suffering from cardiovascular disease. Molnupiravir proved to be highly effective in patients with an incomplete vaccination scheme [115,116].

## 7. Discussion

Treatment of a COVID-19 patient requires interdisciplinary management. Prompt clinical and paraclinical investigations are necessary for the best patient status assessment, enabling the possibility of more individualized care. However, uncertainties arise around the optimal timing of thromboprophylactic therapies, as well as the necessity of these pharmacological interventions, especially in the outpatient setting; some studies contradict each other regarding the clinical outcome of these patients. Chinese guidelines recommend a thrombosis risk assessment for COVID-19 outpatients with underlying vascular pathologies, while others do not recommend pharmacological prophylaxis for any COVID-19 outpatient [117,118,119,120].

A study conducted in Romania, analyzing the effects of the pandemic on the incidence and type of surgical procedures in vascular surgery cases, and providing a comparison with the pre-pandemic period, showed a 34.51% decrease in the overall procedures performed in the pandemic period in this vascular surgery unit. An 80.6% decrease in acute venous insufficiency cases, and a 67.21% increase in acute arterial ischemia were found compared to the pre-pandemic period; furthermore, all the procedures that were not emergencies were postponed, or alternative treatments were used [121]. Unfortunately, the enrollment of the subjects was based on the clinical diagnosis of the acute event, with no mention of the underlying comorbidities, so there is no information on the exact number of acute ischemia or venous insufficiency patients with T2DM as a comorbidity. However, based on another study conducted by the same authors during the pandemic, it can be estimated that this group comprised approximately 40% of all cases [122].

A retrospective study from the Central Ohio Trauma Center involving a total of 260 T2DM patients admitted to the foot and ankle surgery unit showed higher numbers of urgent surgeries and amputations of all kinds during the pandemic versus the pre-pandemic period, and the risk of major amputations increased. The severity of infections also increased in patients with diabetes-related foot problems.

Even with the adoption of telemedicine, home health visits, and reduced in-person clinic hours, medical care was significantly disrupted.

We speculate that the increased severity of diabetic foot infections and major amputations could be linked to an abrupt interruption of, and limited access to, diabetic foot-wound care and limb preservation, as well as patients’ perception of the safety of care during the COVID-19 pandemic [123].

Although venous thromboembolism occurs frequently, and contributes to mortality in COVID-19 patients, arterial thrombosis has also been described (coronary arteries, brain, mesenteric and aortoiliac thrombosis). In a study conducted in the USA, the investigators reported an elevated number of cases presenting lower-extremity ischemia and severe arterial thrombosis during the period of the pandemic. This research reveals an association between lower-extremity arterial thrombosis and SARS-CoV-2 infection (mostly proximal vessels). A higher incidence of death and amputation in COVID-19 patients was described [124].

Despite the administration of anticoagulant medication, the risk of arterial thrombosis continued to rise compared to previous years. The systemic coagulopathy in COVID-19, including the release of inflammatory cytokines, thrombotic events, and microangiopathy, is considered a multifactorial manifestation. Arterial thrombosis, which is less common than venous thrombosis, affected mostly critically ill patients [125].

In a single-center study on the relationship between the coagulation profile and morbidity/mortality in COVID-19 patients performed by A. E. Abd El-Lateef et al., the D-dimer was shown to be a less powerful parameter to predict disease severity and overall survival, compared to FVIII, the von-Willebrand factor antigen, and ristocetin cofactor activity. The ristocetin cofactor activity, alongside the D-dimer and FVIII, independently predicted the disease severity. In the same study, the authors reported reduced survival (30.3%) in patients with higher FVIII levels, and the risk of mortality was extremely (16-fold) increased. Measuring these parameters would help a clinician to decide the therapeutic approach for these patients, aiding in an improvement in disease severity, and in the overall patient survival rates. However, the high costs of these assays are a powerful limitation worldwide, especially in underdeveloped countries [126].

Complications pertaining to the vascular system, such as the cytokine storm that precipitates DIC, and thrombotic microvascular injury affecting the medium- and small-size vessels (coronary heart disease, lung thromboembolism, stroke, mesenteric ischemia, renal-artery thrombosis, and limb-artery thrombosis) are characteristic of COVID-19. Cutaneous lesions associated with arterial and venous thrombotic events appear as gangrene of the limbs [127].

COVID toes or chilblain-like lesions are cutaneous manifestations of COVID-19, especially in pediatric patients, on a background of vascular lesions due to microthrombosis and endothelial inflammation, which might occur in COVID-19, but also after vaccination against SARS-CoV-2. Steroid treatment (systemic, followed by topical therapy) has been proven to be effective in this dermatological disorder [128]. Corticotherapy is also recommended in acute disseminated encephalomyelitis, a life-threatening, immune-mediated neurological complication of COVID-19 [129].

A new, promising perspective for COVID-19 therapy is a combination of anticoagulants and antidepressant drugs (selective serotonin reuptake inhibitors—SSRIs). SSRIs have proven beneficial in the treatment of SARS-CoV-2 infection, by preventing cytokine release [130].

## 8. Originality and Limitations of the Paper

The originality of this review lies in the fact that the authors also included articles published in Hungarian, Romanian, German, and Spanish, besides the English literature, ensuring a wide dissemination of the data obtained. As well as scholarly articles, certain recommendations from national and international guides were also included.

A limitation is the poor consensus on the management of severe COVID-19 cases in different countries, the discrepancy between the available resources of the medical units dealing with these cases, and the lack of broad worldwide experience regarding viscoelastic methods, which represent a further challenge for a comprehensive review on this subject and the formulation of recommendations.

## 9. Conclusions

Inflammation- and infection-induced cytokine storm, vasculopathy, overweight, hyperglycemia, and old age are important risk factors for severe outcomes of COVID-19, involving a hypercoagulable state. Early intervention and proper management could reduce the number of severe cases and the hospitalization time for coronavirus-infected patients with comorbidities.

## Figures and Tables

**Table 1:** Short description of viscoelastic tests. EX-test, extrinsic test; IN-test, intrinsic test; FIB-test, fibrinogen-test; HI-test, heparin test; tPA-test, tissue plasminogen activator test; AP-test, aprotinin-test; RVV-test: Russel viper venom test; ECA test: ecarin (saw-scaled viper venom) test [52].

Test	Reagents Used	Assessed Mechanism	Diagnosis
EX-test	Tissue factor (TF), CaCl_2_, polybrene (to inactivate heparin therapy)	Potential and dynamics of clot formation during tissue damage	Factor deficiencies of extrinsic pathway
IN-test	phospholipid, ellagic acid, CaCl_2_	Potential and dynamics of clot formation during foreign-body contact	Intrinsic pathway factor deficiencies
FIB-test	TF, Ca^2+^, polybrene, cytochalasin D, GPIIb-IIIa inhibitor	Potential and dynamics of secondary clot formation during tissue damage	Fibrinogen deficiency, factor deficiencies of extrinsic pathway
HI-test	lyophilized heparinase, phospholipid, ellagic acid, CaCl_2_	Intrinsic pathway deficiency	Presence of heparin compared to IN-test
tPA-test	TF, CaCl_2_, polybrene, recombinant tissue plasminogen activator (rtPA)	Potential and dynamics of extrinsic coagulation and fibrinolysis	Diagnosis of pathologic fibrinolysis
AP-test	TF, CaCl_2_, polybrene, aprotinin	Extrinsic and common pathway activation and fibrinolysis inhibition	Hyperfibrinolytic bleeding
RVV-test	Russell’s viper venom, CaCl_2_	Factor X activation, common pathway activation, potential and dynamics of clot formation	Direct-acting oral anticoagulant effect
ECA-test	Saw-scaled viper venom (ecarin)	Prothrombin–thrombin activation, dynamics of clot formation	Direct acting oral anticoagulant effect and antithrombin activity

**Table 2:** Guidelines for the management of obese, diabetic subjects, and patients with cardiovascular disease, in the context of SARS-CoV-2 infection.

Obese Patients	Diabetic Patients	Patients with Cardiovascular Diseases
Patient information about COVID-19 symptoms and algorithms to follow in case of symptom progression—telemedicine		
Prevention of infection/reinfection through vaccination; recommendation of wearing a facial mask in crowds, especially during the outbreaks of newer variants		
Monitoring blood pressure and heart rate, with consecutive therapy initiation/modification according to the findings	Monitoring blood pressure and heart rate with consecutive therapy initiation/modification according to the findings and, additionally, dynamic monitoring of carbohydrate balance (blood glucose, HbA1c, C peptide) and, eventually, acid–base balance	Monitoring blood pressure and heart rate, with consecutive therapy modification according to the findings
Diagnosis and therapy of further comorbidities; cardiometabolic risk reduction		
Ensure the availability of hospitalization and eventual intensive care for an unfavorable course of COVID-19		
Weight-loss strategy modification/initiation including diet and physical activity	Considering treatment administration for diabetes mellitus, depending on the type of DM and the severity of COVID-19 symptoms	Treatment of hypertension and/or heart failure focusing on thromboprophylaxis and secondary prevention of complications in the context of COVID-19
Outpatient care after discharge specifically considering the comorbidities of each patient; ideally, an individualized care would be recommended		

## Data Availability

No new data were created or analyzed in this study. Data sharing is not applicable to this article.
